# Job Satisfaction Among Nurses Working in King Abdul Aziz Medical City Primary Health Care Centers: A Cross-Sectional Study

**DOI:** 10.7759/cureus.33672

**Published:** 2023-01-11

**Authors:** Razaz Wali, Hadeel Aljohani, Murug Shakir, Afnan Jaha, Hayat Alhindi

**Affiliations:** 1 Family Medicine, King Abdulaziz Medical City, National Guard Hospital, Jeddah, SAU; 2 Family Medicine, King Abdullah International Medical Research Center, Jeddah, SAU; 3 Family Medicine, King Saud Bin Abdulaziz University for Health Sciences College of Medicine, Jeddah, SAU

**Keywords:** family medicine, saudi arabia, primary health care, nursing, job satisfaction

## Abstract

Background

Job satisfaction in the nursing field directly impacts the quality of patient care. However, increased work demand puts nurses at a higher risk of job dissatisfaction, which can, in turn, affect their work performance. This study aimed to measure job satisfaction among nurses working in National Guard Primary Healthcare Centers (PHCs) and to determine the different sources of pressure at their workplace.

Methods

A cross-sectional quantitative study was conducted among nurses working in the National Guard PHCs in the Makkah region, Saudi Arabia, in 2022. A validated questionnaire from previous literature was used to evaluate nurses' job satisfaction.

Results

A total of 77 nurses completed the questionnaire, with an overall response rate of 89.5%. While 58% (n=45) of nurses were satisfied, 42% (n=32) were dissatisfied. Approximately half the participants were dissatisfied with the rate of payment (49%, n=38), working hours (47%, n=36), and future chances of promotion (44%, n=34). Moreover, 51% (n=39) of nurses attributed considerable pressure to staff shortage and 44% (n=34) to workload. Furthermore, lower mean satisfaction scores in nurses were significantly associated with their intention to leave their current center (p-value= 0.06). In addition, reduced satisfaction scores were frequently observed among females, singles, those who finished their first nurse training five to 10 years ago, those who had a previous experience outside the Ministry of National Guard Health Affairs (MNGHA), those who had only one to five service years, and the ones who belonged to centers that did not have clearly stated standards and policies for nursing practice. However, these associations were statistically not significant.

Conclusion

Results indicate that nurses' job satisfaction should be improved to decrease nurses' intention to leave their workplace and maintain their optimum performance in patient care. This can be achieved by addressing the sources of dissatisfaction and pressure at work.

## Introduction

Nursing is one of the noblest professions. It requires competence, compassion, and genuine commitment to patient caregiving. Since job satisfaction in the nursing field directly impacts the quality of patient care, this topic remains widely discussed and highly valued. Job satisfaction is “a pleasant or positive emotional state resulting from the assessment of our own work or the experience associated with work” [1]. It results in positive outcomes for both the nurses and the patients; some benefits to the nurses are decreased job stress, turnover, and burnout [2]. As work demand increases, nurses have to accommodate a more significant workload while simultaneously meeting the highest standards of patient care. Working in a high-paced environment with shifting work schedules and continuous responsibilities puts them at a higher risk of job dissatisfaction. This affects the nursing staff and reflects in their duties as frontline health workers [3].

Various studies demonstrated that job satisfaction among nurses might also be affected by demographic factors like age, income, work during shifts, and other professional aspects such as years of experience and education. [4]. Job stress, leaders' management styles, empowerment, nursing autonomy, salary, co-worker interactions, and group cohesion are the fundamental factors impacting job satisfaction [2]. In addition, van der Heijden et al. explored how social support, especially from one's co-workers or direct chief, can heavily impact nurses' satisfaction, playing a role in whether or not they will leave their health institute [5]. According to Maslach et al., burnout is another crucial factor strongly correlated to job dissatisfaction [6]. The Maslach Burnout Inventory (MBI) is the most commonly used instrument for measuring burnout [7]. A high burnout score on the MBI is directly linked with a lack of good health and feeling a sense of ineptitude, leading to lower productivity. All eventually lead to job dissatisfaction [3]. In Saudi Arabia, multiple studies have shown similar results regarding job dissatisfaction. For example, AL-Dossary et al. concluded that pay, fringe benefits, contingent rewards, and operating conditions were the main reasons behind nurses' dissatisfaction [4]. In concordance with AL-Dossary et al., Aljohani KA revealed a low-to-moderate level of job satisfaction among nurses [8]. Another study found moderate-to-high levels of burnout and low levels of job satisfaction among critical care nurses [9]. Due to the weight of this topic and the lack of in-depth analysis in Jeddah, it is crucial to investigate it. This study evaluates nurses' satisfaction in National Guard Primary Healthcare Centers (PHCs) in Jeddah, Saudi Arabia. This study aimed to measure job satisfaction among nurses working in National Guard Primary Healthcare Centers (PHCs) and to determine the different sources of pressure at their workplace through a self-administered questionnaire.

## Materials and methods

This is a cross-sectional quantitative study conducted in the National Guard Primary Healthcare Centers in the Makkah region, Saudi Arabia, in 2022. The included centers were Iskan clinic, Specialized polyclinic, Bahraa, and Shareae Primary Healthcare Center. The inclusion criteria for the study were nurses who are currently practicing at said PHCs. The sample size was calculated using the Raosoft sample size calculator, and recommended minimum sample size was 71 participants [10]. Moreover, 86 participants were enrolled in this study, and convenience sampling was the followed sampling technique. Scientific approval was obtained from King Abdullah International Medical Research Center (KAIMRC), and ethical approval was obtained from the Institutional Review Board (IRB) (approval number NRJ21J-155-06). 

The data were collected through a self-administered validated English questionnaire adapted from previous literature [11]. Cronbach's alpha was used to measure the reliability and internal consistency of the questionnaire and run the questionnaire for a pilot study that included participants who would not participate in the main study. The questionnaire has been validated, and Cronbach's alpha of the paper in which it was validated was 0.89 [11]. The questions were mainly given options, with the respondent checking one option. The scales used in the questionnaire are various. Job satisfaction scale: a five-point Likert type scale (1=very dissatisfied, 5=very satisfied), Organizational Commitment Scale: a five-point Likert type scale (1=strongly disagree, 5=strongly agree), Nurses' Occupational Stress Scale: a five-point Likert type scale (1=no pressure, 5=extreme pressure), Professional Identification Scale: a five-point Likert type scale (1=never, 5=very often, and Role Conflict and Ambiguity Scale: a five-point Likert type scale (1=never, 5=very often) [11]. The questionnaire consists of a demographic section followed by sections A, B, C, and D. Section A is mainly about the nurse's perspective on their current job, from different aspects such as pay, job security, and dynamics in the work field. Section B is about the work environment and hospital regulations and policies. Section C is mainly about the sources of pressure at work and obstacles nurses may face. Section D is about how nurses feel about their chosen profession regarding feelings of belonging and support among colleagues [11] (Appendix 1).

Data were entered and analyzed using IBM SPSS Statistics Version 22 (IBM Corp., Armonk, NY). The responses for section A regarding current job satisfaction were merged into three categories (Very dissatisfied/Dissatisfied - Neither satisfied nor dissatisfied - Very satisfied/Satisfied). Similarly, section B regarding the views about their PHC center was also combined into three categories (Strongly disagree/Disagree - Neither disagree nor agree - Strongly agree/Agree). In section C the responses for sources of pressure at work were also categorized into three categories (No/Slight pressure - Moderate pressure - Considerable/Extreme pressure). Section D collecting responses regarding the respondents’ feelings about their chosen profession was also combined into three categories (never/Seldom - Sometimes - Often/Very often). These and the other categorical variables are presented in the descriptive statistics as frequencies and percentages. 

The satisfaction mean scores were calculated by taking the mean of the 15 statements in section A on the five-point Likert scale (1=Very dissatisfied to 5=Very satisfied). These and other numerical variables are presented as mean + standard deviation. The mean satisfaction scores were compared between the categorical variables having two categories using the Independent samples t-test and using ANOVA for variables having more than two categories. A p-value less than 0.05 was considered to show a statistically significant difference for all the statistical tests.

## Results

Demographics

A total of 77 nurses completed the questionnaire, with an overall response rate of 89.5%. The majority of participants were males (70%, n=54), and 45% (n=35) of nurses were 30-44 years old. In addition, most of the respondents (79%, n=61) were either married or divorced, and 49% (n=38) had 1-3 children. Moreover, 64% (n=49) of nurses were Saudi, and the rest were from other countries including the Philippines (23.4%, n=18), Jordan (6.5%, n=5), Egypt (3.9%, n=3), Tunisia (1.3%, n=1), and India (1.3%, n=1). Furthermore, most participants (91%, n= 70) held a Bachelor's degree, and only 9% (n=7) had higher degrees like a Master's degree and Doctorate (Table 1). 

**Table 1:** Demographics.

Demographics		n	%
Gender	Male	54	70%
	Female	23	30%
Age	20 to <30 yrs	24	32%
	30 to <45 yrs	35	45%
	50+ yrs	18	23%
Marital Status	Single	16	21%
	Married / Divorced	61	79%
How many children do you have?	No children	24	31%
	1 to 3 children	38	49%
	>3 children	15	19%
Country of origin	Saudi Arabia	49	64%
	Other	28	36%
Educational level	Bachelor's degree	70	91%
	Other	7	9%

Nurse training 

Most participants chose nursing as their first career choice (82%, n=63). Forty-five percent (n=35) of participants finished their first nurse training more than ten years ago. Additionally, 38% (n=29) of respondents had 1-5 service years, 35% (n=27) had more than 10 service years, and 86% (n= 66) were program-hired. Moreover, more than half of the participants (57%, n=44) had a previous experience outside the Ministry of National Guard Health Affairs (MNGHA). Furthermore, 43% (n=33) of nurses had worked in their current PHC for less than five years, and 38% (n=29) had spent more than ten years working in the same PHC they were attending. However, 48% (n=37) of nurses had the intention to leave their current PHC. Moreover, nearly half of the nurses (48%, n=37) were paid less than 10,000 SR (Saudi Rial) per month (Table 2).

**Table 2:** Nursing training. MNGHA: Ministry of National Guard Health Affairs

Nursing Training		n	%
When did you finish your first nurse training (Year)?	< 5 years ago	14	18%
	5 to 10 years ago	28	36%
	>10 years ago	35	45%
How long have you worked in this primary health care center? (years)	< 5 years	33	43%
	6 to 10 years	15	19%
	> 10 years	29	38%
Do you have a previous experience outside MNGHA?	Yes	44	57%
	No	33	43%
How long are your service years?	1 to 5 years	29	38%
	6 to 10 years	21	27%
	>10 years	27	35%
What is your service type?	Program Hire	66	86%
	SANG / Other	11	14%
What is your income (including basic and extra)?	< 10,000 SR	37	48%
	> 10,000 SR	40	52%
Was nursing your first choice of career?	Yes	63	82%
	No	14	18%
Do you have an intention to leave your current center?	Yes	37	48%
	No	40	52%

As shown in Table 3, team nursing, in which an assigned registered nurse (leader) delegates tasks to a team of medical professionals who care for multiple patients, was the most commonly used patient care delivery system in PHCs (61%, n=43). In addition, 55% (n=35) of nurses did not have/did not know if their PHC had individualized written nursing care plans for each patient. However, 76% (n=51) of nurses reported having nursing notes written at the end of each shift. Moreover, 72% (n=52) of respondents agreed that their PHCs had standardized nursing care plans for common nursing care problems/nursing diagnoses. Furthermore, most participant nurses (87%, n=67) reported that their PHCs had clearly stated standards and policies for nursing practice, and 82% (n=63) revealed that some of these standards and policies were stated by MNGHA. In addition, when asked whether the MNGHA had any regulatory power over nurses, 70% (n=54) of them answered yes. 

**Table 3:** Health center guidelines.

Health Centre Guidelines		n	%
What patient care delivery system is used in your center?	Functional Nursing	17	24%
	Team Nursing	43	61%
	Primary Nursing	10	14%
Do you have individualized written nursing care plans for each patient in your center?	Yes	29	45%
	No / Don't Know	35	55%
Do you have standardized nursing care plans for common nursing care problems/nursing diagnoses in your center? (Procedures and protocols)	Yes	52	72%
	No / Don't Know	20	28%
Do you have nursing notes written at the end of each shift for each patient?	Yes	51	76%
	No	16	24%
Does your primary health care center have clearly stated standards and policies for nursing practice?	Yes	67	87%
	No / Don't Know	10	13%
Are there any clearly stated standards and policies for nursing practice by the Ministry of National Guard Health Affairs?	Yes	63	82%
	No / Don't Know	14	18%
Does the Ministry of National Guard Health Affairs have any regulatory power over nurses?	Yes	54	70%
	No / Don't Know	23	30%

Current job satisfaction

**Figure 1:**
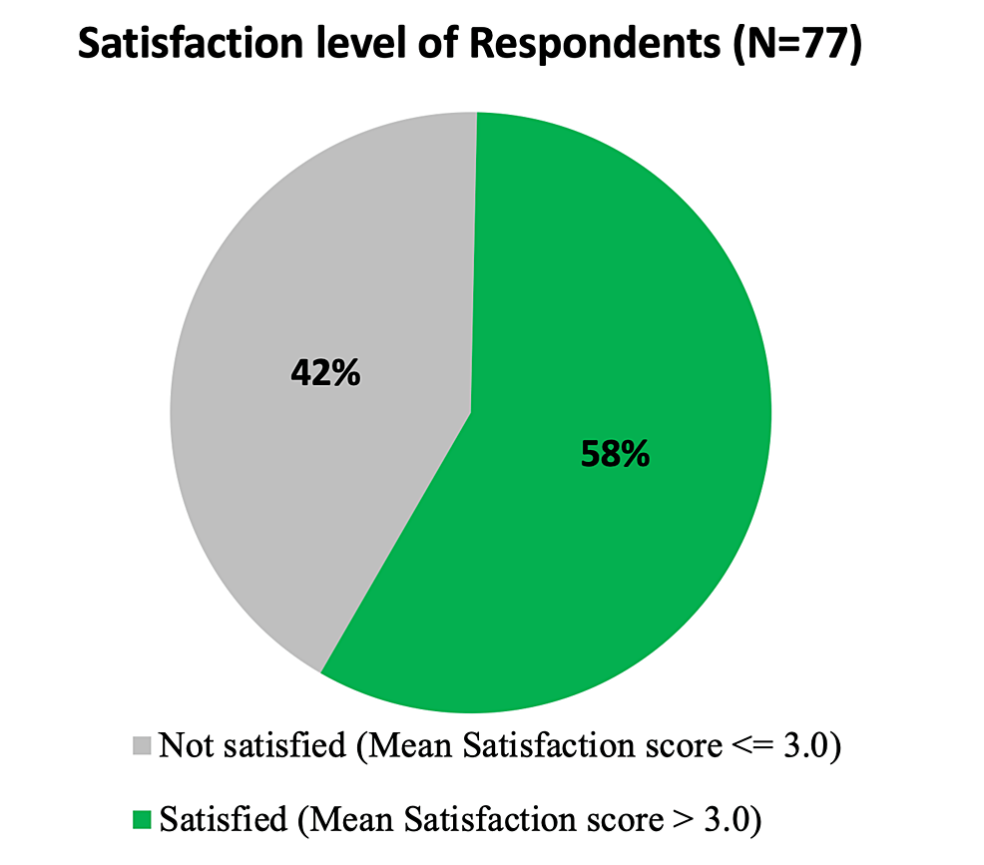
The overall satisfaction level of respondents.

Figure 1 illustrates the overall satisfaction level of respondents. While 58% (n=45) of nurses were satisfied, 42% (n=32) were dissatisfied. The Mean±SD of the satisfaction score was measured to be 3.07± 0.93, where 1 represented the minimum score and 5 represented the maximum. In addition, scores of 3 or less were consistent with dissatisfaction. Details are shown in Table 4.

**Table 4:** Satisfaction mean score (range 1 to 5).

N	77
Mean	3.07
Median	3.33
Std. Deviation	0.93
Minimum	1.00
Maximum	5.00

Table 5 demonstrates nurses' views about their current job satisfaction. Approximately half the participants (49%, n=38) were dissatisfied with the payment rate. In addition, 47% (n=36) of respondents found their working hours dissatisfactory. Moreover, 44% (n=34) of nurses were not satisfied with their future chances of promotion. While 42% (n=32) of participants found how the PHC was managed to be satisfactory, a comparable percentage (39%, n=30) found it dissatisfactory. Furthermore, 45% (n=35) of nurses were satisfied with the attention paid to their suggestions and their job security, while 32% (n=25) were dissatisfied and 36% (n=28) were insecure about their jobs. While 21% (n=16) of respondents were not satisfied with both their immediate bosses and fellow workers, 68% (n=52) and 64% (n=49) were satisfied with their fellows and immediate bosses, respectively. Moreover, slightly more than half of the participants were pleased with the relations between management and staff, the amount of variety in their job, and the amount of responsibility they were given, 53% (n=41), 53% (n=41), and 51% (n=39) respectively).

**Table 5:** Current job satisfaction.

Section A) This section measures nurses' views about their current job satisfaction	Dissatisfied	Neither satisfied nor dissatisfied	Satisfied
The physical conditions in which you work	14	14	49
	18%	18%	64%
Freedom to choose your own working methods	19	20	38
	25%	26%	49%
Your fellow workers	16	9	52
	21%	12%	68%
The recognition you get for good work	25	16	36
	32%	21%	47%
Your immediate boss	16	12	49
	21%	16%	64%
The amount of responsibility you are given	20	18	39
	26%	23%	51%
The rate of pay for nurses	38	17	22
	49%	22%	29%
The opportunity to use your abilities	17	15	45
	22%	19%	58%
Relations between management and staff	21	15	41
	27%	19%	53%
Future chance of promotion	34	23	20
	44%	30%	26%
The way the primary health care center is managed	30	15	32
	39%	19%	42%
The attention paid to your suggestions	25	17	35
	1	22%	45%
The hours of work	36	11	30
	47%	14%	39%
The amount of variety in your job	19	17	41
	25%	22%	53%
Your job security	28	14	35
	36%	18%	45%

Current primary health care center

Table 6 displays nurses' perceptions and attitudes toward the PHCs they were working in. Seventy-four percent (n=57) of participants were willing to put in a great deal of effort beyond what is normally expected to help their center succeed. In addition, 62% (n=48) of respondents agreed that they care about the fate of their center, and 61% (n=47) felt proud to tell others that they were part of their current center. Nevertheless, 52% (n=40) of the nurses could just as well be working for a different center as long as the work was similar, and 40% (n=31) felt very little loyalty to their present center. While 58% (n=45) of nurses were happy that they chose their current center to work for over others they were considering when they joined, 21% (n=16) thought that deciding to work for their current center was a definite mistake. Moreover, 38% (n=29) of participants agreed that there was not too much to be gained by sticking with their current center indefinitely. 

**Table 6:** Current primary health care center.

Section B) This section measures nurses' views about the primary health care center they were working in	Disagree	Neither agree nor disagree	Agree
I am willing to put in a great deal of effort beyond what is normally expected to help this center succeed.	6	14	57
	8%	18%	74%
I talk up this center to my friends as a great organization to work for.	19	18	40
	25%	23%	52%
I feel very little loyalty to this center.	23	23	31
	30%	30%	40%
I would accept almost any type of job assignment in order to keep working for this center.	12	20	45
	16%	26%	58%
I find that my values and this center's values are very similar.	16	26	35
	21%	34%	45%
I am proud to tell others that I am part of this center.	13	17	47
	17%	22%	61%
I could just as well be working for a different center as long as the type of work was similar.	18	19	40
	23%	25%	52%
This center inspires the very best in me in job performance.	20	18	39
	26%	23%	51%
It would take very few changes in my present circumstances to cause me to leave this center.	18	23	36
	23%	30%	47%
I am extremely glad that I chose this center to work for over others I was considering at the time I joined.	13	19	45
	17%	25%	58%
There's not too much to be gained by sticking with this center indefinitely.	23	25	29
	30%	32%	38%
Often, I find it difficult to agree with this center's policies on important matters relating to its employees.	27	27	23
	35%	35%	30%
I really care about the fate of this center.	9	20	48
	12%	26%	62%
For me, this is the best of all possible centers for which to work.	19	21	37
	25%	27%	48%
Deciding to work for this center was a definite mistake on my part.	36	25	16
	47%	32%	21%

Sources of pressure at work

Table 7 shows nurses' viewpoints regarding different sources of pressure at work, ranging in severity from slight to considerable stress. Approximately half of the respondents (51%, n=39) attributed considerable pressure to staff shortage and 44% (n=34) to workload. Additionally, 36% (n=28) of participants thought that dealing with challenging patients caused moderate pressure levels. Moreover, 69% (n=53) of nurses regarded slight pressure to cope with new technologies, and 64% (n=49) to exposure to death. Furthermore, employment security, coping with new situations, and lack of specialized training for present work resulted in slight pressure on more than half of nurses (55%, n=42). In addition, uncertainty about the area of their responsibility was a contributing factor to slight pressure in 51% (n=39) of respondents. Moreover, 48% (n=37) of participants agreed that little pressure was also caused by time pressures and deadlines, tasks outside of their competence, lack of support from senior staff, and lack of participation in planning/decision-making. 

**Table 7:** Sources of pressure at work.

Section C) 1. Sources of pressure at work			
Section C-1) This section measures nurses' views regarding the sources of pressure at work	Slight pressure	Moderate pressure	Considerable pressure
Time pressures and deadlines	37	21	19
	48%	27%	25%
Workload	24	19	34
	31%	25%	44%
Work underload (needing to look busy)	37	19	21
	48%	25%	27%
Task outside of my competence	37	21	19
	48%	27%	25%
Fluctuations in workload	30	26	21
	39%	34%	27%
Unrealistically high expectations of my role	30	24	23
	39%	31%	30%
Coping with new situations	42	17	18
	55%	22%	23%
Uncertainty about the area of my responsibility	39	17	21
	51%	22%	27%
Security of employment	42	12	23
	55%	16%	30%
Involvement with life and death situations	39	19	19
	51%	25%	25%
Coping with new technology	53	13	11
	69%	17%	14%
Exposure to death	49	11	17
	64%	14%	22%
Staff shortages	18	20	39
	23%	26%	51%

2. Sources of pressure at work			
Section C-2) This section measures nurses' views regarding the sources of pressure at work	No or Slight pressure	Moderate pressure	Considerable to Extreme pressure
Poor physical working conditions	35	20	22
	45%	26%	29%
Lack of support from senior staff	37	19	21
	48%	25%	27%
Lack of privacy	38	15	24
	49%	19%	31%
Shortage of essential resources	31	20	26
	40%	26%	34%
Poor quality of supporting staff	34	19	24
	44%	25%	31%
Unsocial hours	36	19	22
	47%	25%	29%
Lack of specialized training for present work	42	14	21
	55%	18%	27%
Lack of participation in planning/decision making	37	21	19
	48%	27%	25%
Difficult patients	20	28	29
	26%	36%	38%
Dealing with relatives	26	21	30
	34%	27%	39%
Bereavement counselling	34	26	17
	44%	34%	22%

Feelings about the nursing profession

Table 8 shows nurses' feelings about their chosen profession. Seventy-one percent (n=55) of participants considered the nursing profession important. In addition, 66% (n=51) of respondents were glad to belong to the nursing profession. Moreover, 48% (n=37) of nurses often felt strong ties with other members of the same profession. However, 19% (n=15) of nurses were annoyed that they were members of the nursing profession, and 18% (n=14) often tried to hide their belonging to the nursing profession. Furthermore, 43% (n=33) of participants sometimes felt held back by the nursing profession.

**Table 8:** Feelings about the nursing profession.

Section D) This section describes nurses' feelings about their chosen profession	Never or Seldom	Sometimes	Often to Very Often
I am a person who identifies strongly with the nursing profession.	7	22	48
	9%	29%	62%
I am a person who makes excuses for belonging to the nursing profession.	35	24	18
	45%	31%	23%
I am a person who feels held back by the nursing profession.	26	33	18
	34%	43%	23%
I am a person who considers the nursing profession to be important.	5	17	55
	6%	22%	71%
I am a person who criticizes the nursing profession.	33	19	25
	43%	25%	32%
I am a person who is glad to belong to the nursing profession.	8	18	51
	10%	23%	66%
I am a person who sees myself as belonging to the nursing profession.	10	17	50
	13%	22%	65%
I am a person who is annoyed to say that I am a member of the nursing profession.	45	17	15
	58%	22%	19%
I am a person who tries to hide belonging to the nursing profession.	47	16	14
	61%	21%	18%
I am a person who feels strong ties with other members of the nursing profession.	15	25	37
	19%	32%	48%

Association between different dependent variables and nurse satisfaction

Table 9 displays that lower satisfaction scores were frequently observed among females and those who were single, even though this was not statistically significant. Moreover, higher satisfaction scores were often seen among those aged 30 to 44 years than in other age groups, although this was not statistically significant. 

**Table 9:** Association between different dependent variables and nurse satisfaction. ^a^ p-value determined using Independent samples t-test ^b^ p-value determined using ANOVA

		Satisfaction mean score (Range 1 to 5)			
Demographics		n	Mean	sd	p-value*
Gender	Male	54	3.15	0.86	0.29^a^
	Female	23	2.90	1.08	
Age	20 to <30 yrs	24	2.91	1.00	0.25^b^
	30 to <45 yrs	35	3.27	0.77	
	50+ yrs	18	2.91	1.09	
Marital Status	Single	16	2.95	0.96	0.57^a^
	Married / Divorced	61	3.10	0.93	
How many children do you have?	No children	24	3.06	0.85	0.99^b^
	1 to 3 children	38	3.09	1.03	
	>3 children	15	3.07	0.85	
Country of origin	Saudi Arabia	49	3.07	0.94	0.99^a^
	Other	28	3.08	0.93	
Educational level	Bachelor's degree	70	3.05	0.92	0.49^a^
	Other	7	3.30	1.10	

Table 10 demonstrates that reduced mean satisfaction scores in nurses had a borderline significance associated with their intention to leave their current center (p-value= 0.06). 

**Table 10:** Association between different dependent variables and nurse satisfaction. ^a^ p-value determined using independent samples t-test ^b^ p-value determined using ANOVA MNGHA: Ministry of National Guard Health Affairs

		Satisfaction mean score (Range 1 to 5)			
Nursing Training		n	Mean	sd	p-value
When did you finish your first nurse training (Year)?	< 5 years ago	14	3.18	1.00	0.60^b^
	5 to 10 years ago	28	2.93	.88	
	>10 years ago	35	3.15	.96	
How long have you worked in this primary health care center? (years)	< 5 years	33	3.05	.91	0.90^b^
	6 to 10 years	15	3.00	1.14	
	> 10 years	29	3.13	.86	
Do you have a previous experience outside MNGHA?	Yes	44	2.98	.98	0.32^a^
	No	33	3.20	.86	
How long are your service years?	1 to 5 years	29	2.94	1.06	0.45^b^
	6 to 10 years	21	3.27	.87	
	>10 years	27	3.07	.83	
What is your service type?	Program Hire	66	3.04	.95	0.43^a^
	SANG / Other	11	3.28	.79	
What is your income (including basic and extra)?	< 10,000 SR	37	3.04	1.07	0.74^a^
	> 10,000 SR	40	3.11	.79	
Was nursing your first choice of career?	Yes	63	3.06	.94	0.84^a^
	No	14	3.12	.93	
Do you have an intention to leave your current center?	Yes	37	2.86	.93	0.06^a^
	No	40	3.27	.90	

Table 11 shows that lesser satisfaction scores were frequently seen among nurses in PHCs that mainly used Primary Nursing, in which a primary registered nurse is assigned to patients to take responsibility for their care throughout the hospital stay, although it was not statistically significant. Additionally, reduced satisfaction scores were frequently observed among those who belonged to centers that did not have individualized written nursing care plans for each patient, centers that did not have standardized nursing care plans for common nursing care problems/nursing diagnoses, and centers that did not have clearly stated standards and policies for nursing practice, and centers that did not have any clearly stated standards and policies for nursing practice by the MNGHA even though these associations were statistically not significant.

**Table 11:** Association between different dependent variables and nurse satisfaction ^a^ p-value determined using independent samples t-test ^b^ p-value determined using ANOVA

		Satisfaction mean score (Range 1 to 5)			
Health Centre Guidelines		n	Mean	sd	p-value
What is the patient care delivery system used in your center?	Functional Nursing	17	3.20	.97	0.80^b^
	Team Nursing	43	3.07	.87	
	Primary Nursing	10	2.95	1.19	
Do you have individualized written nursing care plans for each patient in your center?	Yes	29	3.15	.98	0.32^a^
	No / Don't Know	35	2.91	.93	
Do you have standardized nursing care plans for common nursing care problems/nursing diagnoses in your center? (Procedures and protocols)	Yes	52	3.17	.89	0.19^a^
	No / Don't Know	20	2.85	.99	
Do you have nursing notes written at the end of each shift for each patient?	Yes	51	3.01	1.01	0.96^a^
	No	16	3.00	.82	
Does your primary health care center have clearly stated standards and policies for nursing practice?	Yes	67	3.13	.96	0.18^a^
	No / Don't Know	10	2.70	.66	
Are there any clearly stated standards and policies for nursing practice by the Ministry of National Guard Health Affairs?	Yes	63	3.10	.95	0.65^a^
	No / Don't Know	14	2.97	.89	
Does the Ministry of National Guard Health Affairs have any regulatory power over nurses?	Yes	54	3.02	.94	0.48^a^
	No / Don't Know	23	3.19	.92	

## Discussion

The nursing profession is considered a cornerstone in the healthcare system, and their input matters the most when it comes to patient care. Job satisfaction is an essential aspect of the nursing population's work performance [12]. This study aimed to explore the levels and factors influencing this crucial aspect. In general, it was found in this study that participants had moderate job satisfaction levels, which is similar to what has been found in studies carried out in Oman and Kuwait by Al Maqbali MA and Al-Enezi et al. respectively [12,13]. Findings of studies done in other countries with different cultures and backgrounds also had comparable job satisfaction levels to this study [14,15]. 

Similar to AL-Dossary et al. findings, salary and working hours were identified as sources of dissatisfaction for nurses in this study [4]. This study showed that almost half of the nurses are paid less than 10,000 Saudi Riyals\ 2,666 US Dollars per month, which could explain their dissatisfaction with the payment rate. However, Burnard et al. argued that as important as income seems to influence job satisfaction, it constitutes only a tiny part compared to other factors [16]. Moreover, increased working hours can be caused by staff shortage, which was identified as a source of pressure by the respondents. Nurses might need to take on additional working hours and duties to compensate for this shortage and complete their tasks. As Aljohani described, this pressure creates a poor working environment that exposes nurses to chronic fatigue, poor physical performance, and inefficient communication [8].

While findings of several previous studies showed a significant relationship between levels of job satisfaction and nurses' intention to leave their current centers, the association in this study was borderline significant (p-value= 0.06) [17,18]. Even though it has been found that most participants have a great sense of belonging and loyalty to their workplace and more than half of participants reported that they are satisfied with co-workers, including their bosses, nearly half of them had the intention to leave their centers. Yarbrough et al. linked career development with the nurses' decision on whether to remain or leave their jobs, which, among other factors, could explain the participant's intention to leave in this study since some dissatisfaction with the future opportunities for promotion was found [17]. In addition to job satisfaction, Lu et al. regarded burnout as a contributing factor in retaining nurses and ensuring the quality of care [11]. Future studies are needed to explore nurse turnover and determine the specific aspects related to its rate.

While demographic factors such as age, level of education, and years of experience were positively associated with job satisfaction in previous studies, these associations were not clear or statistically significant in this study [19,20]. An expansion in the sample size could better reveal such relationships since it is considered modest compared to other studies.

Strengths and limitations

The main strengths of this study are the high response rate. Also, the questionnaire used for data collection included different validated scales to measure our outcome. The limited sample size is considered the main limitation of this study. It was determined based on the available study population and may explain why none of the results were statistically significant. Additionally, the generalizability of the results may be limited since the study sample only involves nurses working at National Guard PHCs in Jeddah, Saudi Arabia. Also, working conditions, shift durations, and workload may vary for nurses working in secondary or tertiary healthcare centers compared to PHCs. Therefore, future studies involving a wider range of healthcare facilities would provide a more comprehensive view of the issue. 

## Conclusions

Based on the results demonstrated in this study, many nurses showed dissatisfaction in various areas like rate of payment, working hours, and future chances of promotion. Moreover, sources of nurses' pressure at work varied between the shortage of staff and the amount of workload. Furthermore, reduced job satisfaction may have stimulated nurses to intend to leave their centers. Due to nurses' vital role in the patients' lives, nurses' sources of job dissatisfaction and pressure at work must be addressed and managed.

Recommendations

Healthcare facilities and decision-makers can use the findings of this study to explore the possible changes that can be implemented to improve working conditions for nurses and increase their satisfaction rates and retention. These changes can include improving the work environment, shorter working time, and more financial benefits. Moreover, giving the nurses the opportunity for self-development and more involvement in research activities and international nursing conferences will play a significant role. Also, opening the field for fellowships for those with special interests can make the nursing job more attractive than before. In the future, we would recommend qualitative studies that can help in an in-depth analysis of the current situation and come up with solutions that can increase satisfaction among nurses working in Saudi Arabia or internationally.

## Appendices

Questionnaire 

**Table 12:** Section A The instruction given to the respondent: In this section, I am interested in your views about your current job. For each item, please tick the appropriate box.

Items	Very dissatisfied	Dissatisfied	Neither satisfied nor dissatisfied	Satisfied	Very satisfied
The physical conditions in which you work					
Freedom to chose your own working methods					
Your fellow workers					
The recognition you get for good work					
Your immediate boss					
The amount of responsibility you are given					
The rate of pay for nurses					
The opportunity to use your abilities					
Relations between management and staff					
Future chance of promotion					
The way the hospital is managed					
The attention paid to your suggestions					
The hours of work					
The amount of variety in your job					
Your job security					

**Table 13:** Section B The instruction given to the respondent: In this section, I am interested in your views about working in this hospital. For each item, please tick the appropriate box.

Items	Strongly disagree	Disagree	Neither disagree or agree	Agree	Strongly agree
I am willing to put in a great deal of effort beyond that is normally expected in order to help this hospital be successful.					
I talk up this hospital to my friends as a great organization to work for.					
I feel very little loyalty to this hospital.					
I would accept almost any type of job assignment in order to keep working for this hospital.					
I find that my values and this hospital’s values are very similar.					
I am proud to tell others that I am part of this hospital.					
I could just as well be working for a different hospital as long as the type of work was similar.					
This hospital really inspires the very best in me in the way of job performance.					
It would take very little changes in my present circumstances to cause me to leave this hospital.					
I am extremely glad that I chose this hospital to work for over others I was considering at the time I joined.					
There’s not too much to be gained by sticking with this hospital indefinitely.					
Often, I find it difficult to agree with this hospital’s policies on important matters relating to its employees.					
I really care about the fate of this hospital.					
For me this is the best of all possible hospitals for which to work.					
Deciding to work for this hospital was a definite mistake on my part.					

**Table 14:** Section C The instruction given to the respondent: In this section I am interested in your views about sources of pressure at work. For each item please tick the appropriate box.

Items	No pressure	Slight pressure	Moderate pressure	Considerable pressure	Extreme pressure
Time pressures and deadlines					
Workload					
Work underload (needing to look busy)					
Task outside of my competence					
Fluctuations in workload					
Unrealistically high expectations by others of my role					
Coping with new situations					
Uncertainty about the degree or area of my responsibility					
Security of employment					
Involvement with life and death situations					
Coping with new technology					
Exposure to death					
Staff shortages					
Poor physical working conditions					
Lack of support from senior staff					
Lack of privacy					
Shortage of essential resources					
Poor quality of supporting staff					
Unsocial hours					
Lack of specialized training for present work					
Lack of participation in planning/decision making					
Difficult patients					
Dealing with relatives					
Bereavement counselling					

**Table 15:** Section D The instruction given to the respondent: In this section, I am interested in your feelings about your chosen profession. For each item, please tick the appropriate box.

Items	Never	Seldom	Sometimes	Often	Very often
I am a person who identifies strongly with the nursing profession.					
I am a person who makes excuses for belonging to the nursing profession.					
I am a person who feels held back by the nursing profession.					
I am a person who considers the nursing profession to be important.					
I am a person who criticizes the nursing profession.					
I am a person who is glad to belong to the nursing profession.					
I am a person who sees myself as belonging to the nursing profession.					
I am a person who is annoyed to say that I am a member of the nursing profession.					
I am a person who tries to hide belonging to the nursing profession.					
I am a person who feels strong ties with other members of the nursing profession.					

**Table 16:** Section E The instruction given to the respondent: In this section, I am interested in your experiences at work. For each item, please tick the appropriate box.

Items	Never	Seldom	Sometimes	Often	Very often
I have to do things that should be done differently.					
I receive an assignment without the manpower to complete it.					
I have to buck a rule or policy in order to carry out an assignment.					
I work with two or more groups who operate quite differently.					
I receive incompatible requests from two or more people.					
I do things that are likely to be accepted by one person and not accepted by others.					
I receive an assignment without adequate resources and materials to execute it.					
I work on unnecessary things.					
I feel certain about how much authority I have.					
I have clear, planned goals and objectives for my job.					
I know that I have divided my time properly.					
I know what my responsibilities are.					
I know exactly what is expected of me.					
I get clear explanations of what has to be done.					

**Table 17:** Section F

I. Meeting Physical Needs of Patients Below is a list of care activities which may be performed in the ward to meet the physical needs of patients. Please read each statement carefully and: Firstly, I am interested to know your opinion as a nurse on WHO should mainly carry out this activity. Please read each statement carefully and indicate whether you think it is the role of the staff nurse, health care assistant, doctor, patient’s family or others (e.g. dietician, pharmacist, cleaner etc.). You may tick more than one box per statement if you feel this is a shared activity. Please tick at least one box for each activity. Secondly, please indicate how strongly you feel this is the STAFF NURSE’S role in your current place of practice i.e. always, sometimes, rarely or never by putting a circle around the corresponding number.												
Activities	Staff nurse	Health care assistant	Doctor	Patient’s family		Others	Staff nurse’s role					
							Always		Some-times	Rarely		Never
Attending to patients’ personal cleansing needs when indicated							1		2	3		4
Attending to patients’ dressing needs when indicated							1		2	3		4
Ambulating patients post-operatively							1		2	3		4
Encouraging patients to rest when condition indicates							1		2	3		4
Moving patients who are bedridden regularly to prevent complications							1		2	3		4
Assisting patients with elimination e.g. taking to toilet, giving bedpan and urinal							1		2	3		4
Inserting urinary catheters							1		2	3		4
Removing urinary catheters							1		2	3		4
Carrying out urine testing on the ward							1		2	3		4
Monitoring bowel habits of patients who are at risk							1		2	3		4
Administering suppositories							1		2	3		4
Administering enemas							1		2	3		4
Weighing patients							1		2	3		4
Assisting patients with eating							1		2	3		4
Inserting naso-gastric tubes							1		2	3		4
Feeding patients via naso-gastric tubes							1		2	3		4
Assessing patients’ dietary intake							1		2	3		4
Monitoring diabetic patients’ blood sugar on the war							1		2	3		4
Ensuring patients receive an appropriate diet							1		2	3		4
Administering intravenous fluids as prescribed							1		2	3		4
Checking and adjusting rates of intravenous infusions							1		2	3		4
Charting fluid intake and output when indicated							1		2	3		4
Carrying out venous cannulation							1		2	3		4
Extracting blood by venepuncture							1		2	3		4
Administering prescribed medications							1		2	3		4
Administering prescribed oxygen therapy							1		2	3		4
Taking vital signs							1		2	3		4
Identifying physical signs in patients which are due to illness or treatment							1		2	3		4
Assessing patients for pain							1		2	3		4
Giving prescribed analgesics and assessing their effect							1		2	3		4
Checking and giving prescribed blood transfusions							1		2	3		4
Providing a safe environment for patients and others e.g. dispose of infectious materials and sharps appropriately							1		2	3		4
Dressing wounds aseptically							1		2	3		4
Supervising cleaning of patients’ environment							1		2	3		4
Escorting patients to operating theatre and x- ray department							1		2	3		4
Taking specimens to the laboratory							1		2	3		4
Filing patients’ test results in their notes							1		2	3		4
II. Psychosocial and Communication Aspects of Patient Care Below is a list of activities which reflect psychosocial and communication aspects of patient care. Please read each statement carefully and: Firstly, I am interested to know your opinion as a nurse on WHO should mainly carry out this activity. Please read each statement carefully and indicate whether you think it is the role of the staff nurse, health care assistant, doctor, patient’s family or others (e.g. dietician, pharmacist, cleaner etc.). You may tick more than one box per statement if you feel this is a shared activity. Please tick at least one box for each activity. Secondly, please indicate how strongly you feel this is the STAFF NURSE’S role in your current place of practice i.e. always, sometimes, rarely or never by putting a circle around the corresponding number.												
Activities	Staff nurse	Health care assistant	Doctor	Patient’s family		Others	Staff nurse’s role					
							Always		Some-times	Rarely		Never
Assessing the patient’s educational status prior to providing information												
Informing the patient of his/her diagnosis and prognosis												
Providing health education relevant to the patient’s diagnosis and prognosis												
Encouraging the patient and family to ask questions												
Explaining forthcoming procedures or investigations to the patient												
Discussing with the patient (and carers if necessary) medications which are to be taken after discharge												
Consulting with the patient (and the family if relevant) regarding planned care												
Discussing required care with the family if the patient is going to be dependentfollowing discharge												
Describing concisely and accurately the patient’s condition to other health care teammembers												
Communicating about the patient with other health care team members in writing												
Referring the patient to other health care team members as required												
Reassuring the emotionally upset patient												
Giving information relating to the patient’s condition to the family to minimise anxiety												
III. Professional Aspects of Patient Care Below is a list of activities which reflect professional aspects of patient care. Please read each statement carefully and: Firstly, I am interested to know your opinion as a nurse on WHO should mainly carry out this activity. Please read each statement carefully and indicate whether you think it is the role of the staff nurse, health care assistant, doctor, patient’s family or others (e.g. dietician, pharmacist, cleaner etc.). You may tick more than one box per statement if you feel this is a shared activity. Please tick at least one box for each activity. Secondly, please indicate how strongly you feel this is the STAFF NURSE’S role in your current place of practice i.e. always, sometimes, rarely or never by putting a circle around the corresponding number.												
Activities	Staff nurse	Health care assistant	Doctor	Patient’s family		Others	Staff nurse’s role					
							Always		Some-times	Rarely		Never
Establishing a professional relationship with patients and their family												
Establishing a professional relationship with hospital staff												
Showing respect to patients’ irrespective of age, sex, social status and disease process												
Maintaining patients’ confidentiality												
Exercising accountability in practice												
Maintaining privacy and dignity of the patient												
Complying with hospital regulations and policies related to patient care												
Maintaining a professional attitude throughclean and neat personal appearance and presenting self with appropriate demeanor												
Being aware of ethical guidelines related to patient care												
Acknowledging practice limitations by seeking guidance when needed												
Being aware of drug action doses and side- effects of administered drugs												
Teaching other members of staff												
Updating knowledge and practice skills through continuing education												
Demonstrating practicality in care delivery by operating effectively within resource constraints												
Reporting to a superior circumstances in the ward which could jeopardize standards of care												
Critically reflecting on the practice of self and others in order to improve practice												
Actively seeking continuing professional development to advance knowledge and practice												
IV. Patient Care Management Below is a list of possible activities reflecting aspects of patient care management. Please read each statement carefully and: Firstly, I am interested to know your opinion as a nurse on WHO should mainly carry out this activity. Please read each statement carefully and indicate whether you think it is the role of the staff nurse, health care assistant, doctor, patient’s family or others (e.g. dietician, pharmacist, cleaner etc.). You may tick more than one box per statement if you feel this is a shared activity. Please tick at least one box for each activity. Secondly, please indicate how strongly you feel this is the STAFF NURSE’S role in your current place of practice i.e. always, sometimes, rarely or never by putting a circle around the corresponding number.												
Activities	Staff nurse	Health care assistant	Doctor	Patient’s family		Others	Staff nurse’s role					
							Always		Some-times	Rarely		Never
Assessing the physical status of patients												
Evaluating the signs and symptoms of patients												
Documenting a relevant and detailed assessment of patients’ needs												
Making judgements based on a thorough assessment of all available information												
Diagnosing a range of common conditions												
Writing a plan of care and progress notes for patients												
Implementing planned care												
Assessing patients’ ability to participate in own care i.e. assessing for independence												
Documenting observations relating to changes in condition of patients												
Acting upon evidence of changes in condition of patients												
Supporting colleagues to deliver effective patient care												
Delegating care in a responsible manner												
Coordinating care with other health care team members												
Daily checking and stocking of emergency trolley												
Checking controlled drugs stored in ward												
Being familiar with principles of life support: assessment and maintenance of airway, breathing and circulation												
Initiating cardiac resuscitation												
V. Opinions about Roles of Health Care Personnel The following statements are possible opinions about the roles of staff nurses, health care assistants, doctors and patient’s family members. For each statement please indicate whether you agree, disagree or don’t know by placing a circle around the appropriate number.												
Statement					Agree			Disagree			Don’t know	
There is little if any difference between the range of nursing care activities provided by staff nurses and health care assistants.					1			2			3	
Only doctors should carry out physical assessment of patients e.g. listen to chest and heart sounds.					1			2			3	
Health care assistants should only give basic nursing care designed to meet physical needs of patients like bathing, feeding, dressing and elimination.					1			2			3	
Only staff nurses should give prescribed medications.					1			2			3	
Doctors should be involved in drawing up standards and policies of care for nursing practice.					1			2			3	
Documentation of nursing care is an important part of patient care for staff nurses.					1			2			3	
When a hospitalised patient is dependant in feeding and elimination, a member of his/her family should stay with him/her and help provide this care.												
Doctors should give an order about specific nursing care for their patients.												
Staff nurses should supervise health care assistants.												
Hospital administrators are responsible for the delivery of safe nursing care.												
Doctors should supervise nursing practice and nurses should do what the doctors tell them.												
Staff nurses have adequate knowledge about the action and side effects of the drugs prescribed for patients.												
Senior nurses could teach valuable information to junior doctors.												
Staff nurses can assess the patient’s condition effectively and inform the doctor when required.												
Doctors should tell patients their diagnosis (at first time).												
Each nurse is herself/himself professionally responsible for how well- informed and safe her/his practice is.												
Nurses can decide what nursing care is appropriate for their patients.												
Health care assistants can fully cover the place of staff nurses in his/her absence.												
All nursing staff should comfort and reassure patients.												
Most staff nurses in your hospital are knowledgeable enough to carry out a physical examination.												
The patient’s family solely provides all the emotional support the patient needs.												
Nurses should provide health education for patients.												
Doctors should be responsible for teaching nurses.												

In the following section, I would like to know some information about you and your workplace. Please put an X in the appropriate box 

Age _________ Marital status _________ Educational level _________ 

When did you finish your first nurse training? Year ________ 

How long have you worked in this hospital? __________ Years

Was nursing your first choice of career? Yes ( ) No ( )

If not, please state your first choice of career: __________ 

Do you have an intention to leave your current hospital? Yes ( ) No ( )

What patient care delivery system is used in your ward? (please tick one box which most represents your ward) 

Functional nursing ( )  Team nursing ( )  Primary nursing ( )  Don’t know ( )

In your ward do you have individualised written nursing care plans for each patient? 

Yes ( )  No ( )  Don’t know ( )

In your ward do you have standardised nursing care plans for common nursing care problems/ nursing diagnoses? 

Yes ( )  No ( )  Don’t know ( )

Do you have nursing notes written at the end of each shift for each patient? 

Yes ( )  No ( )  Don’t know ( )

Does your hospital have clearly stated standards and policies for nursing practice? 

Yes ( )  No ( )  Don’t know ( )

Are there any clearly stated standards and policies for nursing practice by the Ministry of Health, P.R. China? 

Yes ( )  No ( )  Don’t know ( )

Does the Ministry of Health, P. R. China have any regulatory power over nurses? 

Yes ( )  No ( )  Don’t know ( )

Thank you very much for taking the time to complete this questionnaire. If you would like to add any information, please do so in the space below. If you have any queries regarding this questionnaire, please contact the researcher Hong Lu on 13701234745. 
